# Associations Between Triglycerides and Walking Capacity in Community-Dwelling Older Adults with Metabolic Syndrome

**DOI:** 10.3390/diseases14010018

**Published:** 2026-01-02

**Authors:** Chiraphat Kloypan, Tichanon Promsrisuk, Boonsita Suwannakul, Chonticha Kaewjoho, Arunrat Srithawong

**Affiliations:** 1 Department of Pathology, School of Medicine, University of Phayao, Phayao 56000, Thailand; chiraphat.kl@up.ac.th; 2 Division of Physiology, School of Medical Sciences, University of Phayao, Phayao 56000, Thailand; tichanon.pr@up.ac.th; 3 Department of Physical Therapy, School of Allied Health Sciences, University of Phayao, Phayao 56000, Thailand; boonsita.su@up.ac.th (B.S.); chonticha.ka@up.ac.th (C.K.)

**Keywords:** metabolic syndrome, triglyceride, walking capacity, older adults, healthy aging

## Abstract

**Background and Objectives**: Metabolic syndrome (MetS) has been associated with reduced physical function in older adults, but the relative contributions of metabolic components, physiological responses, and functional performance to walking capacity remain unclear. **Materials and Methods**: This cross-sectional study included 116 community-dwelling adults aged ≥60 years (mean age 68.5 ± 5.5 years; 65.5% female). Walking capacity was evaluated using the six-minute walk test (6MWT) with associated physiological responses. Functional performance was assessed using the five-times-sit-to-stand test (FTSST), timed-up-and-go (TUG), and handgrip strength. Associations with six-minute walk distance (6MWD) were examined using hierarchical regression analyses, and discriminatory performance was evaluated using receiver operating characteristic analysis. **Results**: Participants with MetS demonstrated shorter 6MWD, slower FTSST and TUG performance, and higher dyspnea ratings compared to those without MetS (*p* < 0.05). Triglycerides were inversely associated with 6MWD in intermediate models (β = −0.33, *p* < 0.001), but after full adjustment, only ΔSBP (β = 0.76, *p* = 0.008) and FTSST (β = −24.45, *p* < 0.001) remained significant. The FTSST and TUG demonstrated excellent discriminatory ability, with AUC values of 0.956 (cut-off ≥ 15.5 s) and 0.925 (cut-off ≥ 13.7 s), respectively, whereas triglycerides showed moderate accuracy (AUC = 0.709) with a cut-off of ≥143 mg/dL. **Conclusions**: Walking capacity was more strongly associated with physiological and functional measures than with metabolic biomarkers. The FTSST and TUG showed strong discriminatory performance for low walking capacity, whereas metabolic markers provided complementary contextual information.

## 1. Introduction

Metabolic syndrome (MetS) is a major global public health concern defined by the coexistence of central obesity, hypertension, dyslipidemia, and impaired glucose regulation. Individuals with MetS are at substantially increased risk of type 2 diabetes and cardiovascular disease and experience significantly higher healthcare utilization and costs [1]. Beyond cardiometabolic morbidity, MetS has also been associated with poorer mental health and reduced health-related quality of life [2]. Current management strategies focus primarily on lifestyle modification, including weight control, dietary improvement, increased physical activity, and smoking cessation, with pharmacological treatment commonly required to manage individual metabolic abnormalities [3]. Despite these evidence-based strategies, the functional consequences of MetS, particularly its impact on mobility in older adults, remain incompletely understood.

Metabolic syndrome (MetS) is increasingly recognized as a multisystem disorder affecting multiple organs, including the heart, liver, vasculature, kidneys, adipose tissue, skeletal muscle, and immune system [4]. Evidence from prior studies indicates that these widespread effects are linked to chronic low-grade inflammation, endothelial dysfunction, hormonal dysregulation, and metabolic disturbances that promote oxidative stress, lipotoxicity, and glucotoxicity [4,5]. Several circulating biomarkers have been proposed to reflect this metabolic and inflammatory milieu, including triglycerides (TG), total cholesterol (TC), low-density lipoprotein cholesterol (LDL-C), high-density lipoprotein cholesterol (HDL-C), C-reactive protein (CRP), leptin, and adiponectin [6]. In individuals with MetS, elevated TG and LDL-C, reduced HDL-C, and increased CRP are commonly observed and are widely interpreted as indicators of metabolic and inflammatory stress [7,8]. Prior literature suggests that such dysregulated biomarker profiles may be associated with endothelial dysfunction, insulin resistance, and impaired lipid metabolism, which may in turn contribute to vascular impairment, hepatic steatosis, and altered skeletal muscle metabolism [9,10,11].

Growing evidence indicates that MetS is associated with impaired physical function beyond its cardiometabolic complications. Previous studies have shown that MetS is linked to subclinical vascular abnormalities [12], elevated oxidative and inflammatory burden [5], frailty, sedentary behavior, and reduced muscle strength [13,14]. Individuals with MetS commonly exhibit poorer mobility, weaker lower-extremity power, reduced balance, and lower cardiorespiratory fitness [15,16,17]. These functional limitations may result from impaired oxygen delivery, neuromuscular inefficiency, and reduced physiological resilience [18,19]. Consequently, older adults with MetS may experience early mobility decline and increased vulnerability to disability.

The six-minute walk test (6MWT) is a widely used and well-validated measure of submaximal exercise capacity in older adults and individuals with cardiometabolic disorders [20]. The 6MWT correlates closely with maximal oxygen uptake and is sensitive to limitations in endurance and overall mobility [21,22]. In addition to reflecting cardiopulmonary capacity, the 6MWT captures functional aspects of daily ambulation and has been shown to identify early mobility decline in older individuals with metabolic abnormalities [23,24]. However, walking capacity is influenced not only by endurance but also by lower-limb strength, balance, and movement control [20,25]. Because walking performance is also influenced by lower-limb strength, balance, and movement control, complementary functional assessments such as the five-times-sit-to-stand test (FTSST), the timed-up-and-go test (TUG), and handgrip strength are commonly used to capture these additional functional domains [26]. Together, these measures provide a more comprehensive assessment of physical function and are practical tools for detecting early functional decline in community-dwelling older adults.

Although previous studies have explored associations between MetS and functional capacity, relatively few have simultaneously examined metabolic components, physiological responses to exertion, and multiple functional performance measures within a single analytic framework [15,16,17]. In addition, existing evidence remains inconsistent regarding whether metabolic biomarkers or functional parameters are more strongly associated with walking capacity in older adults [24,27]. Consequently, the relative contributions of metabolic factors compared with cardiovascular responsiveness and functional performance remain insufficiently defined.

To our knowledge, this study is among the few to concurrently examine the associations between MetS components, physiological responses during the 6MWT, and multiple functional performance measures in community-dwelling older adults. Consequently, the principal objective of this study was to investigate the correlations among MetS components, physiological responses and functional performance, evaluated through 6MWD, FTSST, TUG, and handgrip strength. The secondary aim was to evaluate the discriminatory performance of selected metabolic and functional measures for identifying walking capacity. By integrating metabolic, physiological, and functional domains, this study provides additional evidence to support hypothesis-driven screening approaches for mobility limitation in older adults with metabolic abnormalities.

## 2. Materials and Methods

### 2.1. Participants

This cross-sectional study was conducted at the Dongjen Health Promotion Center, a community-based primary healthcare facility in Phayao Province, northern Thailand. The sample size was calculated using GPower software (version 3.1.9.7) for multiple linear regression analysis. A medium effect size (f^2^ = 0.15) was assumed according to Cohen’s conventions [28], with a two-sided significance level of 0.05 and a statistical power of 0.90. Based on these parameters, a minimum sample size of 116 participants was required. The study protocol was approved by the Institutional Review Board of the University of Phayao (Approval No. HREEC-UP-HSST 1.2/047/68). All participants provided written informed consent before enrollment.

Participants were eligible if they were community-dwelling adults aged 60 years or older, were independent in activities of daily living, and could ambulate independently without walking aids. Exclusion criteria included: (1) musculoskeletal disorders associated with severe pain (e.g., osteoarthritis, gout, rheumatoid arthritis); (2) cognitive impairment, functionally identified as inability to comprehend and execute standardized instructions during the screening interview; (3) recent lower-limb injury or surgery; and (4) uncontrolled hypertension, unstable coronary artery disease, respiratory disease, or active infection.

Participants were classified as having MetS or non-MetS using the updated National Cholesterol Education Program Adult Treatment Panel III (NCEP ATP III) criteria [29]. MetS was defined by the presence of at least three of the following components: (1) waist circumference (WC) ≥ 102 cm in men or ≥88 cm in women; (2) TG ≥ 150 mg/dL; (3) HDL-C < 40 mg/dL in men or <50 mg/dL in women; (4) blood pressure ≥ 130/85 mmHg; and (5) fasting blood glucose (FBG) ≥ 100 mg/dL.

### 2.2. Demographic, Anthropometric, and Metabolic Assessment

Baseline demographic, anthropometric, and metabolic data were collected from all participants. Age and sex were obtained through structured interviews. Body mass index (BMI) was calculated as weight (kg) divided by height squared (m^2^), and WC was measured at the midpoint between the lowest rib and the iliac crest using a non-elastic tape. Physical activity was assessed using the Thai version of the Global Physical Activity Questionnaire (GPAQ, version 2) [30]. GPAQ results were expressed as continuous data in metabolic equivalent (MET)-minutes per week according to World Health Organization guidelines.

For metabolic assessment, after an 8–12 h overnight fast, venous blood samples were collected, and serum was used for biochemical analysis. FBG, TG and HDL-C were measured using enzymatic methods on an automated Sysmex BX-Series chemistry analyzer with commercially available reagents (DiaSys Diagnostic Systems GmbH, Holzheim, Germany) under standard quality control procedures.

### 2.3. Functional Assessments

All functional assessments, including the 6MWT, FTSST, TUG, and handgrip strength, were administered by licensed physical therapists with over five years of clinical experience. Functional-test assessors were blinded to participants’ metabolic and laboratory data to minimize measurement bias.

6MWT: Functional exercise capacity was assessed using the 6MWT conducted along a 30 m indoor corridor. Participants were instructed to walk as far as possible within six minutes without running [20]. Before and immediately after the test, systolic blood pressure (SBP), diastolic blood pressure (DBP), heart rate (HR), and peripheral oxygen saturation (SpO_2_) were measured using an automated sphygmomanometer (Omron Health Care, Kyoto, Japan) and a pulse oximeter (Nellcor™, Pleasanton, CA, USA). Perceived dyspnea and leg fatigue were rated on the modified Borg scale (0–10). The total 6MWD was recorded, and physiological responses were expressed as the change between pre- and post-test values (ΔSBP, ΔHR, ΔSpO_2_).

FTSST: Lower-limb strength and balance were assessed using the FTSST. Participants sat on a 43 cm armless chair with arms crossed and completed five sit-to-stand cycles as quickly as possible without arm support. Timing started at “go” and ended upon sitting after the fifth stand, with total time recorded using a stopwatch [31].

TUG: Mobility and dynamic balance were assessed using the TUG. Participants started seated in the same chair and, on the word “go,” stood up, walked three meters at a comfortable pace, turned around, walked back, and sat down. The total time to complete the task was recorded using a stopwatch [32].

Handgrip strength: Upper-limb strength was assessed using a handgrip dynamometer (TKK 5001; Takei Scientific Instruments, Niigata, Japan). Participants stood with the dominant arm in a neutral position and performed a maximal isometric contraction for about 3 s. Three trials were performed with 15 s rest intervals, and the highest value was recorded for analysis [33].

### 2.4. Statistical Analysis

All analyses were performed using Stata version 14.0 (StataCorp LLC, College Station, TX, USA). The normality of continuous variables was assessed using the Shapiro–Wilk test. Descriptive statistics are presented as mean ± standard deviation (SD) for continuous variables and as frequency (%) for categorical variables. Between-group comparisons (MetS vs. non-MetS) were conducted using appropriate statistical tests based on data distribution: independent *t*-tests for normally distributed variables (e.g., BMI, WC, SBP, DBP, 6MWD), Mann–Whitney U tests for non-normally distributed variables (e.g., FBG, HDL-C, TG, FTSST, TUG, dyspnea, physical activity), and chi-square (χ^2^) tests for categorical variables. Because sex distribution differed significantly between groups, analysis of covariance (ANCOVA) was additionally conducted for functional and physiological performance outcomes (6MWD, FTSST, TUG, handgrip strength, dyspnea, and leg fatigue), with sex included as a covariate.

Associations between TG levels and metabolic, physiological, and functional outcomes were examined using Spearman’s rank correlation. Simple and multiple linear regression analyses were then performed with TG as the independent variable. All adjusted models included age, sex, BMI, and physical activity as covariates due to their known influence on metabolic and functional outcomes. Results are presented as standardized regression coefficients (β) with 95% confidence intervals (CI). To identify determinants of walking capacity, hierarchical multiple regression analysis was conducted using 6MWD as the dependent variable. Model 1 included demographic and anthropometric variables (age, sex, BMI, physical activity). Model 2 added metabolic biomarkers (TG, HDL-C, FBG). Model 3 further incorporated physiological responses (ΔSBP, ΔHR, ΔSpO_2_) and functional performance (FTSST). Because FTSST and TUG were highly correlated (r = 0.864, *p* < 0.001), only FTSST was retained to avoid multicollinearity and redundancy. All regression models were evaluated for linearity, normality of residuals, homoscedasticity, and multicollinearity using residual plots and variance inflation factors (VIFs). Receiver operating characteristic (ROC) curve analyses were performed to assess the discriminatory ability of TG, FTSST, and TUG for walking capacity [34]. The area under the curve (AUC) with 95% CI, optimal cutoff values, sensitivity, specificity, and Youden’s J index were calculated. A *p*-value < 0.05 was considered statistically significant.

## 3. Results

### 3.1. Participant Characteristics

A total of 174 community-dwelling older adults were approached and screened for eligibility at community health centers. Following the screening process, 58 individuals were excluded due to cognitive impairment (*n* = 6), mobility-limiting conditions (*n* = 9), incomplete laboratory data (*n* = 30), or other reasons including declining participation or medical contraindications identified during preliminary assessment (*n* = 13). The remaining 116 participants were included in the final analysis. Based on metabolic syndrome criteria, 58 participants were classified into the MetS group and 58 into the non-MetS group. Table 1 summarizes the characteristics of the 116 participants. The MetS group had a significantly higher proportion of females than the non-MetS group (77.6% vs. 53.4%, *p* = 0.006), while age did not differ between groups (*p* > 0.05). Compared with individuals without MetS, those with MetS showed higher BMI, WC, SBP, FBG, TG, and TG/HDL-C ratio, as well as lower HDL-C levels (*p* < 0.05). DBP and physical activity levels were comparable between groups, as reflected by similar GPAQ scores (1412 ± 1434 vs. 1307 ± 682 MET-min/week; *p* = 0.594). Additionally, the MetS group exhibited significantly shorter 6MWD, slower FTSST and TUG performance, and higher dyspnea ratings (*p* < 0.05, sex-adjusted ANCOVA). Handgrip strength and leg fatigue did not differ significantly between groups (*p* > 0.05).

### 3.2. Associations Between Triglyceride Levels and Physiological-Functional Outcomes

The correlations showed that TG was positively associated with WC (rho = 0.20, *p* = 0.029) and moderately associated with poorer functional performance, including slower FTSST (rho = 0.41, *p* < 0.001) and slower TUG times (rho = 0.47, *p* < 0.001). TG was also negatively correlated with 6MWD (rho = −0.49, *p* < 0.001) and inversely correlated with ΔSBP (rho = −0.27, *p* = 0.004). Meanwhile, associations with HDL-C, FBG, ΔHR, ΔSpO_2_, handgrip strength, dyspnea and leg fatigue were not statistically significant (*p* > 0.05) (Table 2).

After adjustment for age, sex, BMI, and physical activity, TG levels remained inversely associated with ΔSBP (β = −0.286, 95% CI: −0.467 to −0.105, *p* = 0.003). A positive association with ΔSpO_2_ was also observed (β = 0.224, 95% CI: 0.037 to 0.411, *p* = 0.019), whereas no significant association with ΔHR was found (*p* = 0.261). Regarding functional capacity, higher TG levels were associated with shorter 6MWD (β = −0.382, 95% CI: −0.556 to −0.207, *p* < 0.001), slower FTSST performance (β = 0.291, 95% CI: 0.122 to 0.460, *p* = 0.001), and longer TUG time (β = 0.316, 95% CI: 0.152 to 0.480, *p* < 0.001). TG was also associated with weaker handgrip strength (β = −0.038, 95% CI: −0.063 to −0.013, *p* < 0.001). In contrast, no significant associations were observed for perceived dyspnea (*p* = 0.060) or leg fatigue (*p* = 0.885) (Table 2).

### 3.3. Predictors of Six-Minute Walk Distance

In the hierarchical regression analysis, Model 1, which included demographic and anthropometric variables, explained a small proportion of variance in 6MWD (adjusted R^2^ = 0.050, *p* = 0.035). In this model, female sex was associated with shorter walking distance (β = −27.88, *p* = 0.018), whereas higher physical activity levels (GPAQ) were positively associated with 6MWD (β = 0.012, *p* < 0.05). The addition of metabolic biomarkers in Model 2 significantly improved model fit (adjusted R^2^ = 0.213, *p* < 0.001), with TG emerging as a negative predictor of 6MWD (β = −0.33, *p* < 0.001), while FBG showed a nonsignificant trend (β = −0.34, *p* = 0.071). When physiological (ΔSBP, ΔHR, ΔSpO_2_) and functional (FTSST) variables were included in Model 3, the explanatory power increased markedly (adjusted R^2^ = 0.657, *p* < 0.001). In the final model, ΔSBP was positively associated with 6MWD (β = 0.76, *p* = 0.008), whereas longer FTSST times were strongly associated with shorter walking distance (β = −24.45, *p* < 0.001). In contrast, TG, HDL-C, and FBG were no longer significant predictors after accounting for physiological and functional variables (Table 3). Assessment of model assumptions indicated heteroskedasticity, and robust standard errors were applied. No evidence of multicollinearity was observed (VIF < 2).

### 3.4. Discriminatory Accuracy for Identifying Low Walking Capacity

**Figure 1:**
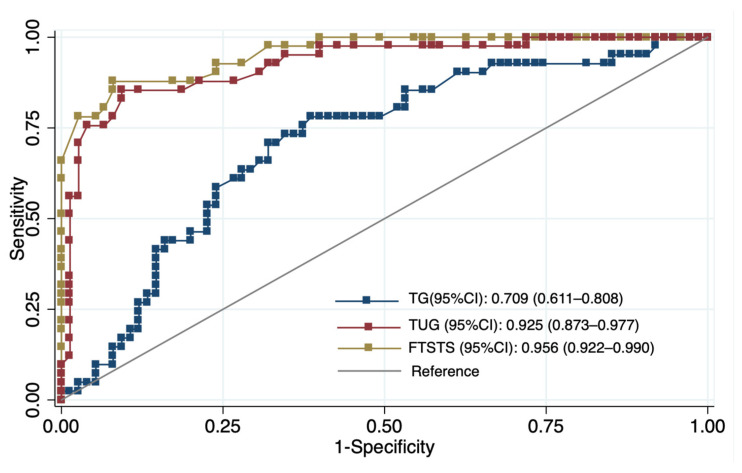
ROC curves demonstrating the discriminative ability of triglycerides (TG), the five-times-sit-to-stand test (FTSST), and the timed-up-and-go test (TUG) to identify low six-minute walk distance (*n* = 116).

The ROC analysis revealed clear differences in discriminatory performance between functional tests and metabolic biomarkers. The FTSST (AUC = 0.956, 95% CI 0.922–0.990) and TUG (AUC = 0.925, 95% CI 0.873–0.977) demonstrated excellent ability to distinguish individuals with low walking capacity. In contrast, triglycerides showed only fair discriminatory performance (AUC = 0.709, 95% CI 0.611–0.808). Based on Youden’s index, the optimal cut-off values were FTSST ≥ 15.5 s (sensitivity 90.20%, specificity 76.00%), TUG ≥ 13.7 s (sensitivity 85.40%, specificity 81.3%), and TG ≥ 143 mg/dL (sensitivity 70.70%, specificity 68.00%) (Figure 1, Table 4).

## 4. Discussion

Our main findings can be summarized as follows. First, older adults with MetS exhibited less favorable metabolic profiles, attenuated cardiovascular responses during the 6MWT, and poorer functional performance than those without MetS. Second, functional measures, particularly FTSST performance and SBP response, emerged as the strongest determinants of 6MWD in adjusted models, while TG levels provided complementary metabolic context. Third, the FTSST showed excellent discriminative accuracy for identifying low walking capacity, while TG demonstrated moderate accuracy and did not independently predict mobility limitation, supporting the use of functional tests as sufficient tools for identifying impaired walking capacity, with TG providing complementary metabolic context.

Our study demonstrated that older adults with MetS exhibited consistently poorer functional capacity—including shorter 6MWD, slower FTSST and TUG performance, and higher dyspnea ratings—compared with those without MetS. These findings align with previous work indicating that MetS is associated with reduced aerobic capacity, slower gait speed and diminished lower-extremity strength [16,17,35]. The higher dyspnea ratings observed in our MetS group may be partly explained by earlier reports suggesting reduced ventilatory and cardiovascular responsiveness during activity, alongside increased metabolic demand and breathing effort [36,37]. Although our study did not directly assess ventilatory mechanics, these theoretical mechanisms are consistent with the broader literature. The poorer FTSST and TUG performance also agree with prior findings linking abdominal obesity, dyslipidemia and impaired glucose regulation to reduced movement efficiency and impaired mobility [15,38,39]. In contrast, handgrip strength did not differ between groups, supporting evidence that upper-limb strength has only a weak association with metabolic risk and mobility [40]. Likewise, the lack of group differences in leg fatigue suggests that subjective perceptions of exertion may be less sensitive than objective lower-limb performance tests, consistent with reports that gait characteristics often remain stable despite experimentally induced fatigue [41].

In regression analyses, TG was initially associated with shorter walking distance, slower functional performance and a blunted SBP response. However, these associations were substantially attenuated in fully adjusted models, and TG demonstrated only moderate discriminatory ability (AUC = 0.709). These results indicate that TG does not function as an independent determinant of 6MWD in this study. Prior research similarly shows that TG lose predictive value for mobility once central obesity, blood pressure and functional performance are considered [16,27]. Studies reporting inverse relationships between the TG/HDL-C ratio and muscle strength [42] further support the interpretation that triglyceride-related dysregulation may relate more to muscle quality than to walking-specific performance. Additional evidence indicates that combinations of MetS components contribute to functional disability [43], reinforcing that metabolic burden is multidimensional rather than driven by TG alone.

The potential mechanism underlying the role of TG in walking capacity in older adults remains unclear. Several biological pathways have been proposed to contribute to functional decline associated with metabolic abnormalities. These include endothelial dysfunction related to dyslipidemia and reduced nitric oxide bioavailability [7,44], intramuscular lipid accumulation and lipotoxicity that impair muscle contractile efficiency [45], and reduced mitochondrial oxidative capacity that limits ATP production and endurance [46,47]. Microvascular dysregulation, including impaired capillary recruitment and restricted oxygen delivery during exertion, has also been documented in individuals with metabolic dysregulation [5,48]. The multisystem effects of metabolic syndrome, such as vascular stiffness, chronic low-grade inflammation, and disrupted muscle vascular crosstalk, may further exacerbate mobility limitations in older adult [27,49]. Hypertriglyceridemia may also impair exercise tolerance through alterations in blood rheology, including increased plasma viscosity and erythrocyte aggregation [50]. Hyperglycemia-induced disturbances in redox balance and oxidative stress may worsen both vascular and muscular function [51,52]. Although these mechanisms were not directly evaluated in the present study, they remain plausible pathways that may contextualize the observed associations and guide future research aimed at clarifying these metabolic effects.

Our hierarchical regression analyses showed that ΔSBP and FTSST were the strongest determinants of 6MWD. These results are consistent with earlier studies emphasizing the importance of cardiovascular responsiveness and lower-limb functional performance in sustaining walking capacity [53,54,55,56]. An adequate rise in SBP during activity reflects preserved autonomic regulation, vascular compliance and the capacity to augment cardiac output [57]. In contrast, the blunted SBP response observed in the MetS group may signify impaired baroreflex sensitivity, increased arterial stiffness or reduced vasomotor responsiveness [58,59]. These cardiovascular abnormalities are well-described consequences of metabolic dysregulation and have been linked to reduced aerobic capacity and diminished exercise tolerance in aging population [37,60].

The discriminative analyses indicate that functional performance measures are the strongest indicators of low walking capacity in older adults, whereas metabolic markers provide complementary contextual information. TG levels demonstrated moderate discriminative ability in ROC analysis (AUC = 0.709), with an optimal cut-off value of 143 mg/dL, yielding balanced sensitivity (70.7%) and specificity (68.0%). This threshold is close to the clinical definition of hypertriglyceridemia (≥150 mg/dL) [61]. However, TG showed weaker performance than functional measures and did not remain independently associated with walking capacity in fully adjusted models, suggesting that metabolic biomarkers alone provide limited explanatory value. Although triglycerides did not enhance discrimination beyond functional tests, modest elevations may reflect underlying cardiometabolic alterations that have been associated in prior studies with reduced skeletal muscle energy metabolism and impaired oxygen utilization [47,62,63,64].

In contrast, functional measures demonstrated excellent discriminative performance. The FTSST (AUC = 0.956, 95% CI 0.922–0.990) and the TUG (AUC = 0.925, 95% CI 0.873–0.977) accurately identified individuals with low walking capacity, with optimal thresholds of 15.5 s and 13.7 s, respectively. These cut-off values are consistent with previous evidence linking prolonged FTSST and TUG times to reduced mobility and increased fall risk among community-dwelling older adults [65,66]. The superior performance of these tests likely reflects their ability to capture integrated functional capacity, including lower-limb strength, balance, coordination, and movement efficiency, which are essential for gait performance and functional independence [67].

Overall, these results indicate that TG, the FTSST and the TUG represent complementary dimensions of mobility limitation in older adults. TG reflects systemic metabolic burden, while FTSST and TUG directly capture neuromuscular and balance-related determinants of walking capacity. Integrating metabolic and functional assessments may therefore enhance early detection of mobility decline. In resource-limited primary care and community settings, combining simple functional tests with routine lipid profiling may provide a practical and cost-effective approach to identify at-risk individuals and guide timely interventions.

This study has several limitations that should be acknowledged. First, the cross-sectional design precludes causal inference between metabolic abnormalities, cardiovascular responses, and functional performance. Second, cognitive status was not formally assessed using standardized neurocognitive instruments; although participants were able to follow test instructions, subtle cognitive impairments that may influence mobility cannot be fully excluded. Third, several clinical and behavioral factors that may affect both metabolic status and mobility, including medication use, habitual diet, objectively measured physical activity, and inflammatory markers, were not comprehensively assessed. The absence of these variables may partly explain the attenuation of triglyceride-related associations in adjusted models. Fourth, dyspnea and leg fatigue were assessed using subjective ratings, which may limit sensitivity for detecting exertional abnormalities. Fifth, although both men and women were included, the study was not powered for sex-stratified analyses, and the modest sample size and single-center recruitment may limit generalizability. Finally, mechanistic physiological measures, such as endothelial function, skeletal muscle composition, mitochondrial activity, and blood rheology, were not directly evaluated. Future longitudinal studies with comprehensive metabolic and physiological assessments are needed to clarify causal pathways linking metabolic dysregulation to mobility decline.

## 5. Conclusions

MetS was associated with reduced walking capacity and poorer functional performance in community-dwelling older adults. TG showed limited independent association with walking capacity, whereas functional measures, particularly the FTSST and systolic blood pressure response during the 6MWT, were the strongest indicators of mobility limitation, supporting the use of simple functional tests for clinical identification of impaired walking capacity.

## Figures and Tables

**Table 1:** Participant characteristics by metabolic syndrome status.

Variable	All (*n* = 116)	Non-MetS (*n* = 58)	MetS (*n* = 58)	*p*-Value
Demographic and anthropometrics				
Female sex, *n* (%)	76 (65.5%)	31 (53.4%)	45 (77.6%)	0.006 *
Age (years)	68.46 ± 5.48	68.74 ± 5.52	68.17 ± 5.48	0.586
BMI (kg/m^2^)	22.49 ± 3.56	21.73 ± 3.70	23.25 ± 3.28	0.021 *
WC (cm)	84.07 ± 9.11	82.09 ± 8.65	86.05 ± 9.20	0.018 *
GPAQ (MET-min/wk)	1359.15 ± 1119.49	1411.76 ± 1434.44	1306.53 ± 682.07	0.594
Cardiometabolic and clinical parameters				
SBP (mmHg)	129.87 ± 17.04	123.71 ± 13.99	136.03 ± 17.68	<0.001 *
DBP (mmHg)	71.47 ± 9.17	70.10 ± 8.99	72.83 ± 9.22	0.110
FBG (mg/dL)	117.55 ± 28.65	105.55 ± 19.11	129.55 ± 31.58	<0.001 *
HDL-C (mg/dL)	44.79 ± 12.33	48.41 ± 13.68	41.17 ± 9.63	0.007 *
TG (mg/dL)	150.47 ± 71.21	120.16 ± 57.56	180.79 ± 71.05	<0.001 *
TG/HDL-C ratio	3.60 ± 1.88	2.66 ± 1.55	4.55 ± 1.71	<0.001 *
Functional and physiological performance ^†^				
6MWD (m)	334.97 ± 60.28	364.44 ± 53.33	305.51 ± 52.15	<0.001 *
FTSST (s)	15.12 ± 1.96	14.48 ± 1.62	15.75 ± 2.08	0.005 *
TUG (s)	13.15 ± 1.85	12.34 ± 1.64	13.96 ± 1.69	<0.001 *
Handgrip strength (kg)	21.89 ± 4.88	22.85 ± 5.17	20.93 ± 4.41	0.388
Dyspnea (Borg 0–10)	2.91 ± 1.58	2.54 ± 1.58	3.28 ± 1.50	0.017 *
Leg fatigue (Borg 0–10)	2.21 ± 1.40	2.29 ± 1.43	2.13 ± 1.38	0.520

Values are presented as mean ± SD for continuous variables and as number (percentage) for categorical variables. * *p* < 0.05 indicates a statistically significant difference between the MetS and non-MetS groups. **^†^** Adjusted for sex using ANCOVA. BMI, body mass index; WC, waist circumference; GPAQ, Global Physical Activity Questionnaire; MET, metabolic equivalent of task; SBP, systolic blood pressure; DBP, diastolic blood pressure; FBG, fasting blood glucose; HDL-C, high-density lipoprotein cholesterol; TG, triglycerides; 6MWD, six-minute walk distance; FTSST, five-times sit-to-stand test; TUG, timed up-and-go test.

**Table 2:** Associations between triglyceride levels and metabolic, physiological, and functional outcomes among community-dwelling older adults (*n* = 116).

Variables	rho (*p*-Value)	β Standardized (95% CI)	*p*-Value
Metabolic and Behavioral profile			
WC (cm)	0.20 (0.029) *	0.059 (−0.102, 0.220)	0.465
HDL-C (mg/dL)	−0.01 (0.939)	−0.049 (−0.249, 0.151)	0.625
FBG (mg/dL)	0.05 (0.622)	−0.036 (−0.214, 0.142)	0.705
Physiological responses (6MWT)			
ΔSBP (mmHg)	−0.27 (0.004) *	−0.286 (−0.467, −0.105)	0.003 *
ΔHR (bpm)	0.09 (0.331)	0.102 (−0.077, 0.281)	0.261
ΔSpO_2_ (%)	0.17 (0.074)	0.224 (0.037, 0.411)	0.019 *
Functional capacity			
6MWD (m)	−0.49 (<0.001) **	−0.382 (−0.556, −0.207)	<0.001 **
Handgrip strength (kg)	−0.11 (0.244)	−0.038 (−0.063, −0.013)	<0.001 **
FTSST (s)	0.41 (<0.001) **	0.291 (0.122, 0.460)	0.001 **
TUG (s)	0.47 (<0.001) **	0.316 (0.152, 0.480)	<0.001 **
Perceptual responses			
Dyspnea (Borg 0–10)	0.17 (0.064)	0.186 (−0.008, 0.380)	0.060
Leg fatigue (Borg 0–10)	−0.00 (0.981)	−0.015 (−0.187, 0.157)	0.885

* Significant at *p*-value < 0.05; ** Significant at *p*-value < 0.001. rho, Spearman’s rank correlation coefficient; β, standardized regression coefficient from multiple linear regression adjusted for age, sex, BMI, and physical activity. Δ variables represent post-pre changes. TG, triglycerides; WC, waist circumference; HDL-C, high-density lipoprotein cholesterol; FBG, fasting blood glucose; SBP, systolic blood pressure; HR, heart rate; SpO_2_, peripheral oxygen saturation; 6MWD, six-minute walk distance; FTSST, five-times-sit-to-stand test; TUG, timed-up-and-go test.

**Table 3:** Multiple regression predicting six-minute walk distance (6MWD) among community-dwelling older adults (*n* = 116).

Predictors	Model 1 β (95% CI)	Model 2 β (95% CI)	Model 3 β (95% CI)
Demographic and Anthropometric			
Age (years)	−0.93 (−3.15, 1.30)	−2.11 (−4.25, 0.03))	0.98 (−0.56, 2.52)
Female (ref: male)	−27.88 (−50.64, −4.93) *	−18.39 (−40.02, 3.24)	15.19 (−0.67, 31.05)
BMI (kg/m^2^)	−2.21 (−5.43, 1.02)	−0.37 (−3.46, 2.71)	−0.02 (−2.10, 2.07)
GPAQ (MET-min/wk)	0.012 (0.0003, 0.023) *	0.007 (−0.003, 0.018)	0.01 (−0.00, 0.01)
Metabolic biomarkers			
TG (mg/dL)		−0.33 (−0.47, −0.18) **	−0.09 (−0.20, 0.03)
HDL-C (mg/dL)		0.02 (−0.79, 0.84)	0.40 (−0.16, 0.96)
FBG (mg/dL)		−0.34 (−0.71, 0.03)	−0.19 (−0.45, 0.06)
Physiological and Functional (6MWT)			
ΔSBP (mmHg)			0.76 (0.20, 1.31) *
ΔHR (bpm)			0.08 (−0.44, 0.60)
ΔSpO_2_ (%)			−0.01 (−5.46, 5.45)
FTSST (s)			−24.45 (−32.44, −16.45) **
Adjusted R^2^	0.05	0.213	0.657
ΔR^2^	-	0.138	0.423
*p*-value	0.035 *	<0.001 **	<0.001 **

* Significant at *p*-value < 0.05; ** Significant at *p*-value < 0.001. β, unstandardized regression coefficient; CI, confidence interval; BMI, body mass index; GPAQ, Global Physical Activity Questionnaire; MET, metabolic equivalent of task; TG, triglycerides; HDL-C, high-density lipoprotein cholesterol; FBG, fasting blood glucose; ΔSBP, change in systolic blood pressure; ΔHR, change in heart rate; ΔSpO_2_, change in oxygen saturation; FTSST, five-times sit-to-stand test; 6MWD, six-minute walk distance. Model 1 includes demographic and anthropometric variables; Model 2 adds metabolic biomarkers; Model 3 incorporates physiological and functional parameters.

**Table 4:** ROC analysis for identifying low walking capacity among community-dwelling older adults (*n* = 116).

Predictor	AUC (95% CI)	Optimal Cut-Off	Sensitivity (%)	Specificity (%)
TG (mg/dL)	0.709 (0.611–0.808)	≥143	70.70	68.00
FTSST (s)	0.956 (0.922–0.990)	≥15.5	90.20	76.00
TUG (s)	0.925 (0.873–0.977)	≥13.7	85.40	81.30

AUC, area under the curve; CI, confidence interval. TG, triglycerides; FTSST, five-times-sit-to-stand test; TUG, timed-up-and-go test.

## Data Availability

The data presented in this study are available on request from the corresponding author. The data are not publicly available due to ethical restrictions related to participant privacy.

## Abbreviations

The following abbreviations are used in this manuscript:

6MWD	Six-Minute Walk Distance
6MWT	Six-Minute Walk Test
AUC	Area Under the Curve
BMI	Body Mass Index
DBP	Diastolic Blood Pressure
FBG	Fasting Blood Glucose
FTSST	Five-Times-Sit-to-Stand Test
GPAQ	Global Physical Activity Questionnaire
HDL-C	High-Density Lipoprotein Cholesterol
HR	Heart Rate
MET	Metabolic Equivalent of Task
MetS	Metabolic Syndrome
SBP	Systolic Blood Pressure
SpO_2_	Peripheral Oxygen Saturation
TG	Triglycerides
TG/HDL-C	Triglyceride-to-HDL Cholesterol Ratio
TUG	Timed-Up-and-Go Test
VO_2_max	Maximal Oxygen Uptake
WC	Waist Circumference
